# Detection of Dihydrocoumarin in Coconut Juice via Photoelectric Detection System Based on Ultraviolet Absorption Spectrometry

**DOI:** 10.3390/s22218267

**Published:** 2022-10-28

**Authors:** Xiaoyan Wang, Jiangyu Xu, Rendong Ji, Haiyi Bian, Xiaotao Feng, Zhezhen Jiang, Xinyue Guo, Yuan Zhang, Ahmed N. Abdalla

**Affiliations:** Jiangsu Laboratory of Lake Environment Remote Sensing Technologies, Huaiyin Institute of Technology, Huai’an 223003, China

**Keywords:** ultraviolet absorption spectrum, photoelectric detection, PSO-SVR, dihydrocoumarin

## Abstract

Based on ultraviolet absorption spectroscopy technology combined with stoichiometry, a portable photoelectric detection system with wireless transmission was designed with the advantages of simple operation, low cost, and quick response to realize the non-destructive detection of dihydrocoumarin content in coconut juice. Through the detection of a sample solution, the light intensity through the solution is measured and converted into absorbance. Particle swarm optimization (PSO) is applied to optimize support vector regression (SVR) to establish a corresponding concentration prediction model. At the same time, in order to solve the shortcomings of the conventional portable photoelectric detection equipment in data storage, data transmission, and other aspects, based on the optimal PSO-SVR model, we used Python language to develop a friendly graphical user interface (GUI), integrating data collection, storage, analysis, and prediction modeling in one, greatly simplifying the operation process. The experimental results show that, compared with the traditional methods, the system achieves the purpose of rapid and non-destructive detection and has a small gap compared with the detection results of the ultraviolet spectrophotometer. It provides a good method for the determination of dihydrocoumarin in coconut juice.

## 1. Introduction

With the rapid development of modern science technology, and economy, and improvements in living standards, people have higher requirements for the quality of food. However, some manufacturers use excessive food additives in order to pursue strong taste, sales, and profit maximization, which are likely to cause great harm to people’s bodies. In order to change this situation, it is necessary to strengthen the research on the detection methods of food additives. The traditional detection methods for food additives mainly include: gas chromatography (GC) [1], high-performance liquid chromatography (HPLC) [2], capillary electrophoresis (CE) [3], liquid chromatography–mass spectrometry (LC-MS) [4], and supercritical fluid chromatography (SFC) [5]. These methods usually have high detection accuracy, but they also have shortcomings in some aspects, such as a generally destructive, difficult, and long sampling period, high cost, complex sample preparation, personnel training requirements, and advanced laboratory requirements. Therefore, it is of great practical significance to develop a method for food additive concentration detection with a simple operation that is low cost and responds quickly.

Ultraviolet absorption spectrometry is commonly used as a detection method for food additives. It has the characteristics of a short measurement cycle, simple operation, and online monitoring, and is suitable for a portable photoelectric detection system [6]. According to the UV absorption characteristics of food additives in the sample solution, this method first obtains the absorbance at the characteristic peak of the absorption spectrum, the corresponding maximum absorbance at a certain wavelength, and then establishes the quantitative relationship between the absorbance and the concentration of food additives. The authors of [7] used ultraviolet spectrophotometry to measure the content of nitrite and nitrate, and the spiked recovery rate was between 98% and 102%. Meanwhile, they studied the concentration of the sample solution, the determination conditions, the influence of interference, and the accuracy and precision of the method for eliminating the influence of interfering substances. The authors of [8] established a detection model of coumarin in cortex fraxini extract based on ultraviolet spectrophotometry, with an average recovery of 100.6% (RSD = 1.8%). The method was simple, accurate, and reliable. The approach presented in [9] realized the quality control of stevia extract in the food industry based on UV/visible spectrophotometry, and this method had very good consistency and acceptable deviation, providing a new way for the quality control of stevia extract. Dihydrocoumarin is a type of food additive, which is widely used in spices, food, cosmetics, agriculture, and other fields. Long-term exposure and excessive consumption will lead to skin allergies, toxic damage to the human liver [10], and even cancer, so the detection of coumarin content is particularly important. The Hygienic Standard for the Use of Food Additives stipulates that the maximum amount of dihydrocoumarin in beverages, cold drinks, and candies is 7.8, 21.0 and 44.0 mg/Kg, respectively [11]. This paper explains the design of a portable wireless transmission system used for photoelectric detection. The system first detects coconut juice containing dihydrocoumarin of different concentrations, reads its absorbance and transmits it wirelessly, then develops a GUI interface based on Python language to process and analyze absorbance data, and finally establishes a quantitative model to realize the concentration prediction and preliminary classification of dihydrocoumarin in coconut juice, and its detection and prediction effects were compared with the ultraviolet spectrophotometer. The error between the two was small, indicating that the system achieved good results.

## 2. Materials and Methods

### 2.1. Materials

Dihydrocoumarin (C_9_H_8_O_2_) (CAS No.: 119-84-6, molecular weight: 148.16, purity: 99%, colorless to pale yellow transparent liquid, Shanghai Maclin Biochemical Technology Co., Ltd., Shanghai, China) was researched as a food additive to detect its absorption spectrum in experiments; dimethyl sulfoxide (DMSO) (C_2_H_6_OS) (CAS: 67-68-5, molecular weight: 78.13, colorless transparent liquid, Sinopharm Chemical Reagent Co., Ltd., Shanghai, China) was used as a solvent to dissolve the dihydrocoumarin samples with 100% coconut juice from fresh coconut.

### 2.2. Experiments

First, add 50 mg dihydrocoumarin to the dimethyl sulfoxide for dissolution, and the standard solution with a mass concentration of 1 mg/mL is obtained after full stirring. Then, pipette 1 mL of pure coconut juice and 2.5 mL of dihydrocoumarin standard solution into a 50 mL measuring cup, and add DMSO to the 50 mL scale line to obtain a sample solution with a concentration of 0.050 mg/mL dihydrocoumarin in the coconut juice. Take 3.4 mL of the sample solution and put it into a 1 cm cuvette for testing. The dilution method is to pour 0.2 mL from the cuvette and add 0.2 mL of DMSO to the cuvette. After each addition, fully stir and mix them to obtain 30 groups of experimental samples, with the corresponding concentration range of 0.009 ~ 0.050 mg/mL. The portable photoelectric detection system is designed to detect and measure the absorbance data of the dihydrocoumarin in coconut juice, which is wirelessly transmitted to the GUI software platform for quantitative modeling and analysis. Finally, the absorbance data obtained after the detection and the corresponding sample concentration value are stored in the MySQL database [12] for future analysis.

### 2.3. System Principle

#### 2.3.1. Detection Principle Based on Ultraviolet Absorption Spectrum

UV absorption spectrometry follows Beer–Lambert law, that is, within a certain absorption light path b (cm), the concentration of absorbent material c (g/L) in the sample is proportional to the absorbance A. The formula is:

(1) $$A=\log\frac{I_{0}}{I}=\log\frac{1}{T}=\mathrm{Kbc}$$

I_0_ is the incident light intensity, I is the transmitted light intensity, T is the transmission ratio, and K (L/(g·cm)) is the absorption coefficient. When the sample solution contains multiple components, the total absorbance equals the sum of the absorbance of each component.

In this study, the concentration of dihydrocoumarin in coconut juice and the corresponding absorbance at the absorption peak of the ultraviolet absorption spectrum is also in accord with the above Beer–Lambert law. Therefore, the quantitative analysis of dihydrocoumarin in coconut juice can be realized by detecting its UV absorption spectrum. On this basis, this paper designs and implements a portable wireless transmission photoelectric detection system.

#### 2.3.2. PSO-SVR

The support vector regression algorithm [13] is used to establish the mathematical model in the UV analysis software in this paper, which was proposed by Vapnik et al. in 1995 and is now widely used. The basic principle is to create a “spacer band” on both sides of the linear function with a spacing of ϵ (tolerance of deviation); no loss is calculated for all samples falling into the interval band, that is, only the support vector will have an impact on its functional model. Finally, the optimized model is obtained by minimizing the total loss and maximizing the interval.

In order to further improve the accuracy of the prediction model, PSO is used to optimize the SVR algorithm [14]. PSO is an optimization algorithm based on “population” and “evolution”. In the process of particle swarm optimization, each particle represents a potential solution of the problem. The fitness value is used to evaluate the quality of the particle. The particle speed is dynamically adjusted according to the historical information of its own movement and the movement experience information of other particles, so as to realize the optimization in the solution space. The parameters such as the C (penalty factor), epsilon, and the coefficient gamma of kernel function of the SVR algorithm are optimized using PSO, and the parameters corresponding to the optimal determination coefficient (R^2^) are selected within a certain range. The best parameters after searching were set to C = 3.987, epsilon = 0, and gamma = 0.514, respectively. In this paper, PSO-SVR is used as the core algorithm to establish the functional model of dihydrocoumarin absorbance and concentration, and realize the quantitative analysis of dihydrocoumarin in coconut juice.

### 2.4. System Design

#### 2.4.1. Hardware Structure

**Figure 1:**
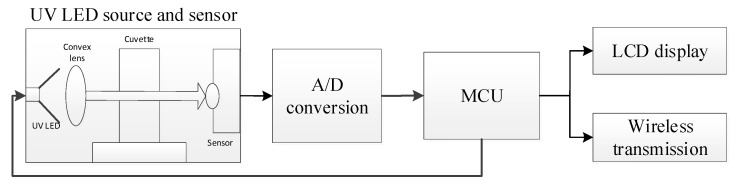
Structure diagram of the portable photoelectric detection system based on wireless transmission.

The portable photoelectric detection system based on wireless transmission mainly consists of an ultraviolet light-emitting diode (LED), a convex lens, a photoelectric sensor, an analog to digital (A/D) conversion module, a microcontroller unit (MCU) minimum system, liquid crystal display (LCD), a wireless transmission module, etc. The UV LED adopts 3535UVC from Shenzhen Gushitai Industrial Co., Ltd., Shenzhen, China, with a central wavelength of 270 nm, a wavelength range of 260~280 nm and the size of 3.5 × 3.5 mm. The convex lens has a concentrating effect on the light, which can prevent the divergence of the light and improve the detection accuracy of the sample. The MUC adopts STC89C52 which is a low-power, high-performance 51 core CMOS 8-bit microcontroller. CJMCU-GUVA-S12SD is selected as the ultraviolet sensor module. The sensor has the advantages of low power consumption, good linearity, and high sensitivity, and its detection range is 240~370 nm with a size of 11 × 28 mm. The wireless transmission module esp8266 [15] is used to transmit the detection data to the host computer instead of the conventional serial data transmission, which makes the use space of the device more flexible and free from the constraints of data lines. The LCD1602 display module is used to display the absorbance value of the test sample. The structure block diagram is shown in Figure 1.

#### 2.4.2. Software Process

**Figure 2:**
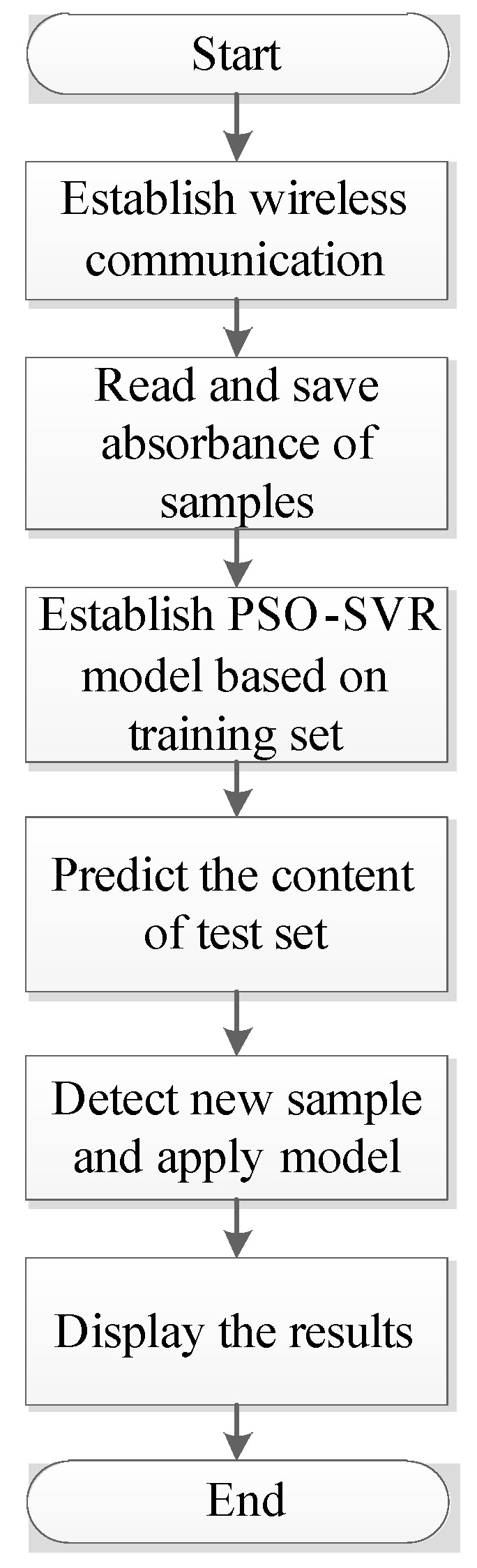
Flow chart of the GUI software for UV absorption spectrum analysis.

At present, many researchers have designed spectral analysis software based on some commonly-used chemometrics algorithms, many of which are developed based on Matlab [16]. Compared with Matlab language software, the application program based on Python language has better portability and is more conducive to the promotion and application of software. This paper designs a GUI interface based on Python language to realize spectrum data display, database storage, and modeling analysis functions. The developed upper GUI software and the hardware module of MCU together constitute a portable photoelectric detection system with wireless transmission, which can realize the measurement of sample absorbance data, wireless transmission, data storage and display, PSO-SVR model training, sample concentration prediction, and other functions. As shown in Figure 2, more details of the GUI software for UV absorption spectrum analysis are described as follows:

(1) Establish wireless communication connection between GUI software and MCU.
(2) Read the absorbance data of all samples (dihydrocoumarin in coconut juice) detected by the portable photoelectric detection system and store them in the MySQL database together with the corresponding concentration values. The data of these samples will be used to establish and validate the PSO-SVR model.
(3) The sample data in the database are divided into the training set and test set according to a certain proportion. The PSO-SVR prediction model is established based on the training set, and the relationship between sample concentration and absorbance is obtained.
(4) The PSO-SVR model is applied to the test set to obtain the prediction results of dihydrocoumarin content in coconut juice, and the output plot shows the relationship between the predicted value and the real value of the dihydrocoumarin content in the test set, so as to evaluate the performance of the PSO-SVR model.
(5) After obtaining a quantitative analysis model with good performance, detect and read the absorbance of dihydrocoumarin in the new coconut juice sample, predict its concentration according to the trained PSO-SVR model, and compare it with the concentration threshold of 0.020 mg/mL set by ourselves based on the maximum use of dihydrocoumarin in food and the detection accuracy of the portable detection system. If the predicted concentration value is lower than this threshold, it is classified as safe, otherwise it is classified as unsafe.

**Figure 3:**
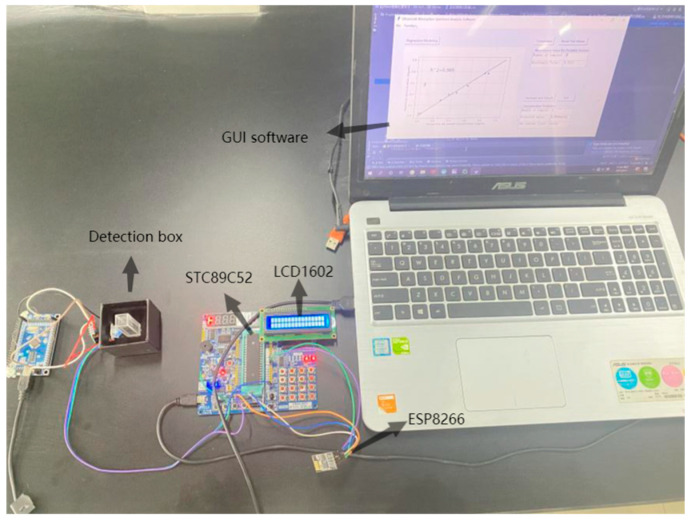
Detection of dihydrocoumarin in sample solutions using the portable photoelectric detection system with wireless transmission.

Figure 3 shows the rapid detection of the dihydrocoumarin sample solution via the portable photoelectric detection system with wireless transmission. The left side is the hardware detection system, which realizes the detection of samples and the wireless transmission of absorbance data. The right side is the upper GUI analysis software, which realizes the functions of data reading, data storage, analysis, and prediction.

## 3. Results and Discussion

### 3.1. Absorption Spectra of Dihydrocoumarin

**Figure 4:**
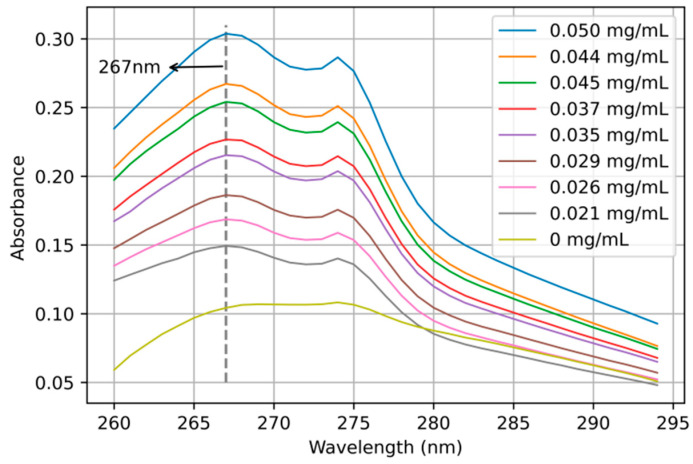
Absorption spectra of dihydrocoumarin in coconut juice at different concentrations.

**Figure 5:**
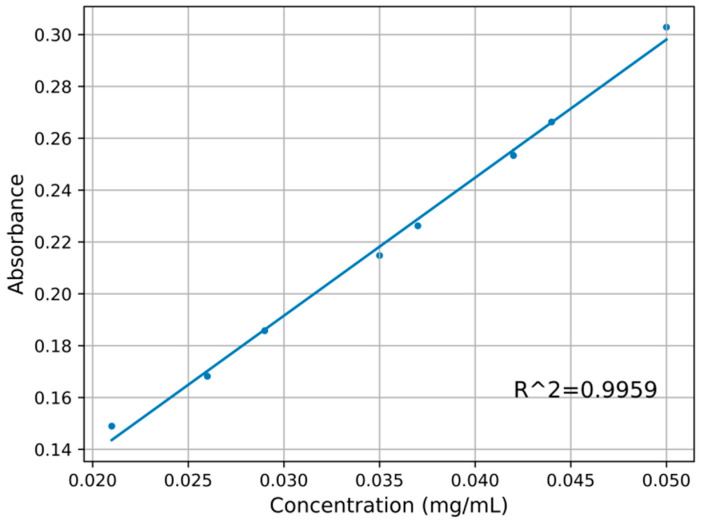
Relationship of dihydrocoumarin concentration and absorbance at 267 nm.

According to the sample preparation method described in Section 2.2, 30 groups of dihydrocoumarin solutions in coconut juice with different concentrations were scanned for absorption spectral characteristics with the UV spectrophotometer (UV6300, Shanghai Meipuda Instrument Co., Ltd., Shanghai, China). The absorption spectra of nine samples are shown in Figure 4. Obviously, compared with dihydrocoumarin, there is no absorption peak in pure coconut juice. By comparing the absorption peaks of coconut juice containing different concentrations of dihydrocoumarin, it was found that the absorption peak of dihydrocoumarin was near 267 nm. The higher the concentration of dihydrocoumarin, the greater the corresponding absorbance value. If we extract the absorbance at 267 nm in Figure 4, the concentration of dihydrocoumarin is linear with the absorbance, as shown in Figure 5. The absorbance of dihydrocoumarin at 267 nm has a strong linear relationship with the concentration of dihydrocoumarin, and the determination coefficient is 0.9959. The error bar obtained through three groups of parallel tests is also shown in Figure 5. It can be seen that it has good reproducibility and low error. Based on the above analysis, the concentration of dihydrocoumarin in the sample solution can be predicted by ultraviolet absorption spectrometry.

### 3.2. Software Performance

**Figure 6:**
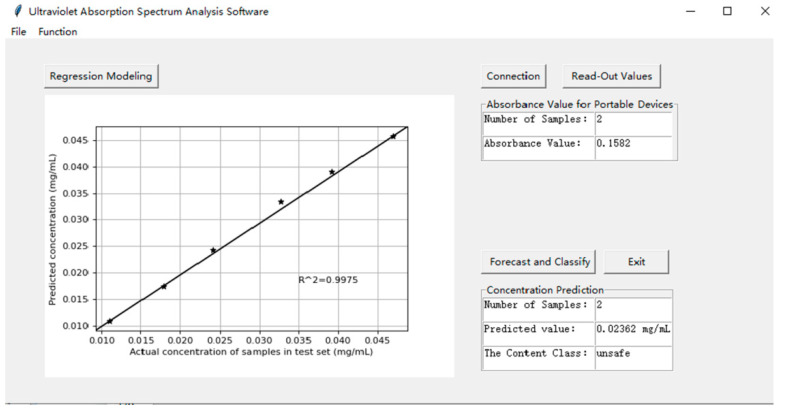
Content prediction results of the test set on GUI platform of portable detection system.

The operation interface of GUI software is shown in Figure 6, which mainly includes two functions: training set modeling and test set content prediction. The left area displays the results of content prediction in graphical form, and the right area displays the absorbance value, predicted concentration value, and preliminary sample classification result of the current test sample in text form.

After the wireless connection between the GUI software and MCU was established, the absorbance values of 30 groups of coconut juice samples obtained via the method described in Section 2.2 were also measured by the self-made portable detection equipment and stored in the MySQL database together with the corresponding concentration values. Among them, 24 groups of samples were selected as the training set, and the remaining 6 groups of samples were selected as the test set. The PSO-SVR concentration prediction model is trained based on the training set on the GUI platform and applied to the six samples of the test set to predict the corresponding concentration values. The specific results are shown in the curve on the left area of Figure 6; the determination coefficient of the fitting result is 0.9975 which shows that the model has good predictability. The right area of Figure 6 shows the relevant information of No. 2 sample whose actual concentration is 0.023 mg/mL, including the absorbance value (0.1582), the predicted concentration value (0.02362 mg/mL), and the classification of the sample (unsafe). The predicted concentration is higher than the threshold value of 0.020 mg/mL, so it is classified as an unsafe category. In addition, based on the slope of the linear fitting curve (6.101) and the standard deviation of the blank sample response value (0.00461), the method validation parameters including limit of detection (LOD) and limit of quantitation (LOQ) were calculated for the dihydrocoumarin detection using the portable device, where LOD was 0.003 mg/mL and LOQ was 0.008 mg/mL.

**Figure 7:**
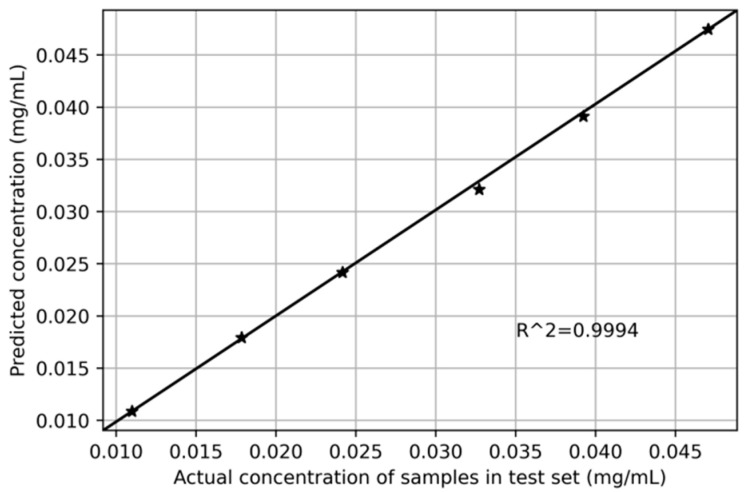
Content prediction results of test set based on UV6300 spectrophotometer.

At the same time, we also used the UV6300 spectrophotometer to scan 30 groups of dihydrocoumarin sample solutions in coconut juice, and selected 24 groups as the training set, and the other 6 groups as the test set, which is the same as the portable detection system. Similarly, we applied the PSO-SVR algorithm to establish a quantitative function model, predict the concentration of dihydrocoumarin in coconut juice, and fit it with the actual concentration value to obtain the relationship between them. As shown in Figure 7, the black asterisk represents six samples of the test set, the horizontal axis shows the actual concentration of dihydrocoumarin, and the vertical axis shows the predicted concentration. The determination coefficient of the fitting result is 0.9994, which indicates that the prediction accuracy of the model based on the data measured by the UV6300 spectrometer is slightly better than that of the self-designed portable detection system.

### 3.3. Recovery and Precision

In order to further verify the accuracy of the PSO-SVR prediction model and the detection effect of the portable photoelectric detection system, UV6300 and portable equipment were used to detect the other three groups of coconut juice samples with different concentrations of dihydrocoumarin. We added the dihydrocoumarin standard solution with the concentration of 0.050 mg/mL into the diluted coconut juice sample to obtain three groups of sample solutions with the concentration of 0.018, 0.023 and 0.035 mg/mL. The recovery rate can be calculated according to the ratio of the predicted concentration value to the actual concentration. For the precision experiment, repeat the analysis six times for each of the three groups of sample solutions under the same conditions, and represent the method precision with the relative standard deviation (RSD). The calculation results are shown in Table 1.

Table 1 shows that when the sample concentration is 0.018 mg/mL, the average recovery corresponding to the portable detection system is 94.61%, RSD is 2.29%, and the average recovery corresponding to the UV6300 spectrophotometer is 97.85% and RSD is 0.33%. When the sample concentration is 0.023 mg/mL, the average recovery and RSD corresponding to the portable system is 101.26% and 1.76%, respectively, and they are 102.34% and 0.375% for the UV6300 spectrophotometer. When the sample concentration is 0.035 mg/mL, the average recovery and RSD of the portable system is 102.64% and 1.65%, respectively, and they are 98.62% and 0.32% for the UV6300 spectrophotometer. This shows that the prediction effect of the PSO-SVR model is good, and the gap between the portable system and the UV6300 spectrophotometer is small, but the accuracy of the portable system needs to be further improved. In addition, among the above three test samples, two samples are classified as an unsafe category according to the predicted concentration value, and one sample is classified to be safe both for the portable system and the UV6300 spectrophotometer. The classified result is also consistent with the result corresponding to the actual concentration.

The portable detection system also has some measurement errors, which mainly come from the following aspects. The light source intensity will fluctuate in a small range, and the light will be more divergent due to the large luminous angle. The light transmittance of the lamp bead lens is 93% due to its material, which is not completely transparent. The sensitivity of the sensor to ultraviolet light of different wavelengths will be slightly different. In addition, it also includes the operational errors in the preparation of experimental samples. The overall system needs to be further improved. While it is necessary to maintain the stability of the light source intensity, select more appropriate lenses to reduce the light source divergence, and improve the transmittance to reduce the measurement error introduced in the hardware system design, it is also necessary to be more rigorous and serious in the actual operation process of sample preparation.

## 4. Conclusions

This paper designs a portable photoelectric detection system based on the ultraviolet absorption spectrum, and develops GUI software based on Python language. On this software platform, the quantitative analysis of dihydrocoumarin in coconut juice was realized by training the PSO-SVR model. The coconut juice samples containing dihydrocoumarin are detected and analyzed based on this photoelectric detection system. The light intensity signal of the samples is measured and converted into the absorbance value which is wirelessly transmitted to the GUI platform to establish the PSO-SVR regression model between the concentration and absorbance of the dihydrocoumarin samples. The PSO algorithm is used to optimize the parameters of the SVR model. From the results of average recovery and RSD in experimental verification, the error of the system is small compared with that of the ultraviolet spectrophotometer, which confirms that the designed system can quickly and nondestructively predict the concentration of dihydrocoumarin in coconut juice and realize the preliminary classification of the concentration level of dihydrocoumarin solution. The designed portable photoelectric detection system based on wireless transmission integrates the functions of absorbance data acquisition, transmission, storage, and quantitative analysis modeling, greatly simplifies the actual operation process, and has the advantages of simple operation, low cost, and fast response, which can be used for the non-destructive detection and content prediction of dihydrocoumarin in coconut juice.

## Figures and Tables

**Table 1:** Comparison of recoveries and precisions of two methods (*n* = 6).

Measuring Method	0.018 mg/mL		0.023 mg/mL		0.035 mg/mL	
	Average Recovery %	RSD %	Average Recovery %	RSD %	Average Recovery %	RSD %
Portable detection system	94.61	2.29	101.26	1.76	102.64	1.65
UV6300	97.85	0.33	102.34	0.375	98.62	0.32

## Data Availability

Data underlying the results presented in this paper are not publicly available at this time but may be obtained from the authors upon reasonable request.
